# Extrinsic Incubation Period of Dengue: Knowledge, Backlog, and Applications of Temperature Dependence

**DOI:** 10.1371/journal.pntd.0002207

**Published:** 2013-06-27

**Authors:** Nils Benjamin Tjaden, Stephanie Margarete Thomas, Dominik Fischer, Carl Beierkuhnlein

**Affiliations:** Department of Biogeography, University of Bayreuth, Bayreuth, Germany; University of California, Davis, United States of America

## Background

Dengue is generally believed to be one of the most hazardous vector-borne diseases, with over 40% of the world's population at risk of an infection [1]. While in the past the disease has mainly been observed in the tropical regions, recent studies suggest that, under the pressure of future climate change, new areas as far north as Europe may become endangered. In fact, in 2010 the first European cases of autochthonous dengue since the epidemic outbreak in Greece in the late 1920s [2] were reported from Croatia [3] and France [4]. Recently, Madeira experienced a severe epidemic of dengue fever, with about 2,000 cases within two months [5].

When it comes to determining the risk of dengue occurring in a given region, the extrinsic incubation period (EIP) plays an important role. The EIP is commonly defined as “the interval between the acquisition of an infectious agent by a vector and the vector's ability to transmit the agent to other susceptible vertebrate hosts” [6]. In the case of dengue, after the virus is ingested by a mosquito through a blood meal, some time is required for the virus to replicate, escape the midgut, and spread through the mosquito's body until it ultimately reaches the salivary glands (SG), from where it can be passed on to another host during the next blood meal.

For dengue, the duration of the pathogen's EIP is known to be temperature-dependent, but very few mechanistic risk models (usually based on the basic reproductive number *R*_0_, i.e., the number of secondary cases produced by one primary case in a completely susceptible population [7]) have taken that into account until now. In fact, most of the models implemented for dengue use fixed values for the duration of the EIP or rather rough estimates of temperature dependence [8].

This may be due to the fact that experimental studies on this topic are rare, and their results may appear to some extent inconsistent or even contradictory. However, the implementation of a realistic, temperature-dependent EIP will greatly improve mechanistic dengue modeling: since EIP appears as an exponent in the equations used for the determination of *R*_0_ and vector capacity [7], [9], [10], even small changes in EIP can have a large impact on the results of mechanistic dengue models that build on the concept of *R*_0_. The practical relevance of this issue has been demonstrated for dengue [9] as well as other vector-borne diseases such as malaria [11] and bluetongue [12].

In addition, correlative models based on environmental factors and vector distributions (also referred to as “climate envelope models” or “environmental niche models”) have to be revised and enhanced. Currently, these models usually focus on the spatial distribution of vector species. But if temperatures do not support amplification and establishment of the virus even though the vector is present, risk assessment based solely on vector distributions leads to an overestimation of areas at risk. Combining such models with information on temperature requirements for the virus derived from the EIP can reduce uncertainty [13].

Here, we give a short overview of the few experimental studies that are explicitly addressing the temperature dependence of the EIP of dengue. We analyze the implications of these studies and discuss current uncertainties in modeling dengue risk in face of climate change. We identify methodological challenges and formulate suggestions for the design of future studies from a spatio-ecological point of view.

## What Has Been Done So Far?

In order to assess current knowledge about the temperature dependence of the EIP of dengue, we conducted an extensive literature search, using the Thomson Reuters Web of Knowledge research portal (which includes the databases Web of Science, BIOSIS, Current Contents Connect, MEDLINE, and Journal Citation Reports) as well as Google Scholar and Google Books. Search terms were built from all possible combinations of the keywords “dengue,” “DENV,” “extrinsic,” “EIP,” “incubation period,” and “temperature.” Journal articles and books that were found to provide secondary information on the topic were scanned for references to experimental studies, and a forward and reverse literature search was performed for experimental studies.

We found five experimental studies that explicitly addressed the temperature dependence of the EIP of dengue. The first one was carried out by Blanc and Caminopetros in Greece during the winter of 1928–1929 [14]. This was followed by two publications by McLean et al. in the mid-1970s [15], [16] and another article by Watts et al. in 1987 [17]. Rohani et al. revived the topic in 2009 [18]. In addition to these works, we include two further studies in the dataset that examine the duration of the EIP at a single, fixed temperature: Salazar et al. [19] studied the spread of dengue virus within the body of *Aedes aegypti* at 28°C, and Anderson and Rico-Hesse [20] examined the effect of viral genotype on the vector capacity of *A. aegypti* at 30°C.

**Figure 1:**
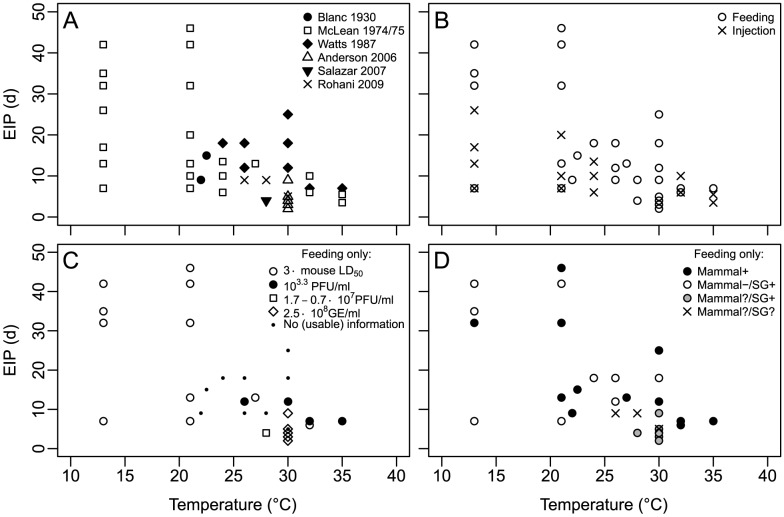
Overview of the available data for the temperature dependence of the EIP of dengue. Each point represents the duration until the first observed transmission or infection of SG at a given temperature in a single experiment. (A) Complete dataset, divided by study. (B) Complete dataset, divided by method used to infect the mosquitoes: results obtained by letting mosquitoes feed on infected mammals or artificial blood meals versus results obtained via intrathoracic injection of virus solution. (C) Data from mosquitoes infected via feeding, divided by the amount of virus ingested by mosquitoes. GE, genome equivalents; LD_50_, mean lethal dose; PFU, plaque forming units. (D) Data from mosquitoes infected via feeding, divided by method of demonstration of transmission. Black circles: Transmission was demonstrated by allowing infected mosquitoes to feed on mammals. White circles: Tests on mammals yielded negative results, but SG contained virus. Grey circles: Tests on mammals were not done, but SG contained virus. Xs: Neither transmission to mammals nor SG were tested.

All experiments have in common that they examined the EIP of dengue virus type 2 in *A. aegypti*, with the exception of Blanc and Caminopetros, who did not provide information about the serotype examined (retrospective studies suggest dengue virus types 1 and 2 occurred during the Greece epidemic [21]), and Rohani et al., who additionally examined dengue virus type 4. However, the experimental approaches vary considerably in many respects within and between the studies. An overview of the durations of the EIP as observed by the different studies is given in Figure 1A; a detailed list can be found in Table S1.

Differences start with the study material used: the provenance of the mosquitoes used ranges from recently captured wild animals [14] to colonies that had been held in the laboratory for more than 30 years [18]. Since populations that have been held in the laboratory for a longer time may develop adaptions to the artificial environment, field-relevant mosquitoes are preferred for determining EIP, in order to yield results that reflect natural processes as closely as possible [19]. This is also true for viruses that have been maintained in the laboratory for longer periods [19]. Additionally, it is highly important to cover the whole range of genetic variations that occur in nature, since it has been demonstrated that different genotypes or strains of the dengue virus can show significant differences regarding their EIP [10], [15], [20].

Moreover, differences in experimental techniques for infecting the mosquitoes became obvious: while intrathoracic injection of virus solution provides the opportunity to exactly determine the amount of virus a mosquito receives, it bypasses the midgut infection and escape barriers. This drastically shortens the EIP [15], [22], leading to overestimation in the process of risk assessment. In the case of dengue, this problem affects about 60% of the data points by McLean et al. [15], [16] (Figure 1B). Hence, we strongly suggest the use of more natural and realistic feeding techniques that use viremic vertebrates or artificial blood meals.

Since the duration of the EIP also depends on the amount of virus ingested during the blood meal, ideally the complete range of virus titers observed in vertebrate hosts in nature should be considered. The methods and units used for determining and presenting the amount of virus differ across the experiments, making it difficult to conduct an adequate comparison (see Figure 1C for an overview and Table S1 for the details). While a consistent methodology would surely help to make the results of such experiments more comparable and more accessible for scientists from other fields, in our eyes the most important issue is to make sure that future experiments resemble nature as closely as possible.

Furthermore, the method used to test the ability of an infected mosquito to transmit the virus should be chosen carefully. Allowing the mosquito to take a second blood meal from uninfected mammals such as mice [15], [16], monkeys [17], or even humans [14], and then monitoring the mammals for dengue symptoms or virus content may seem desirable, since it gives rather clear evidence of transmission. However, because of ethical as well as logistical restraints, in most cases this cannot be considered as an option anymore today. Consequently, other methods have been developed that focus on the detection of virus content in the SG of the mosquito. While it is generally assumed that transmission can occur as soon as the SG are infected, the literature provides some cases where the SG tested positive for virus content but additional transmission tests with mammals gave negative results [15]–[17]. A possible explanation for this may be the existence of a “salivary gland escape barrier,” which has been shown to exist for other viruses [23] but which is considered controversial for dengue [24]. However, new techniques exist that circumvent this potential problem by causing mosquitoes to spill their saliva, which can then be assayed for virus content [22]. Equally, methods that use complete heads or even full bodies to extract virus RNA are not suitable for the assessment of the EIP. The latter method was used by Rohani et al. [18], unfortunately making their data unsuitable for real-life modeling approaches even though the data seem to be consistent with the rest of the dataset. An overview of the implications of this issue for the dataset is presented in Figure 1D; additional details are given in Table S1.

**Figure 2:**
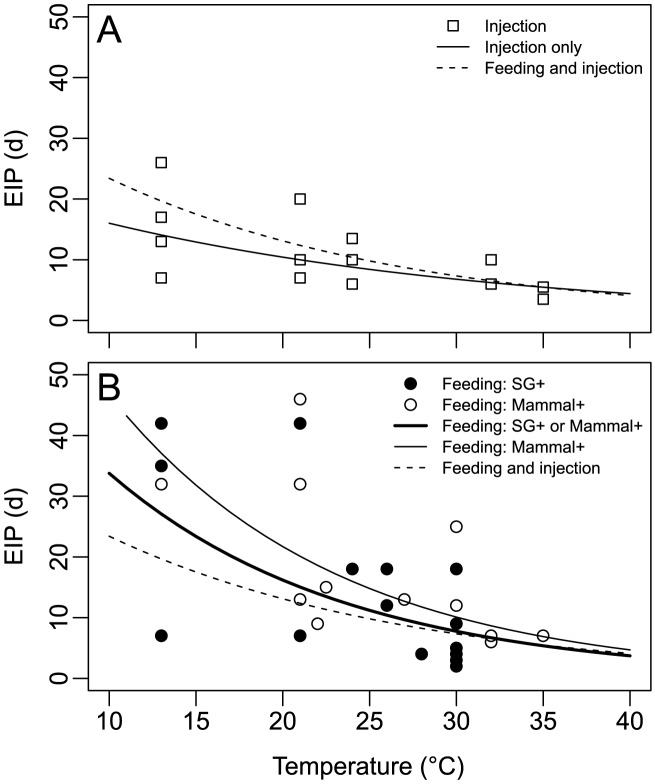
Estimated temperature dependence of the EIP of dengue based on the dataset used. Each point represents the duration until the first observed transmission or infection of SG at a given temperature in a single experiment. Estimation was done via a simple linear model in R 2.14.1 [31], using log-transformed values of the duration of the EIP. (A) Results obtained from experiments with mosquitoes infected via intrathoracic injection; the solid line depicts the linear model for those data (adjusted *R* ^2^ = 0.40, *p*<0.001). (B) Results obtained from experiments with mosquitoes infected via feeding. Filled circles: SG tested positive for virus content, but transmission to mammals was either negative or not tested. Unfilled circles: Transmission to mammals was observed. Thick solid line: Linear model for cases where either transmission to mammals was observed or SG tested positive for virus content (adjusted *R* ^2^ = 0.34, *p*<0.001). Thin solid line: Linear model for cases where transmission to mammals was observed (adjusted *R* ^2^ = 0.46, *p*<0.01) For better comparability, in both panels the dashed line shows the linear model for all data (injection as well as feeding) combined (adjusted *R* ^2^ = 0.32, *p*<0.00001).

Careful preprocessing is crucial in order to gain meaningful results from the data that are currently available. First, experimental results that were obtained using intrathoracic injection to infect mosquitoes should be discarded, since their inclusion would lead to underestimation of the EIP and thus overestimation of areas at risk (Figure 2A). Then, data points for which verification of transmission does not exist by either examination of vertebrates bitten during a second blood meal or by examination of the SG should be discarded, too. Whether one wants to include data points for which transmission was verified only via examination of the SG may depend on the context the data are being used in: Figure 2B shows that the inclusion of these points in general leads to a shorter mean EIP, particularly at the lower end of the temperature range. Hence, risk maps based on a dataset that includes those points may overestimate the threat in regions with lower temperatures—which from an ethical point of view would be preferable to the underestimation that would probably result from the exclusion of those data. Additionally, data obtained from experiments at low temperatures (<20°C) are especially scarce, so that further reduction must be carefully weighed for statistical reasons.

## Design of Future Experiments with Respect to Interdisciplinary Research

Apart from the specific problems that arose in analyzing the experiments that have been done so far, there are some other things that might be worth considering when it comes to planning future works. Because the EIP varies between single mosquitoes, usually a batch of mosquitoes is examined for each time point during the experiment. The EIP can then be estimated as the period of time between the infectious blood meal and the point in time when (1) for the first time at least one mosquito of the batch is able to transmit the virus, (2) a given fraction (typically 50%) of the mosquitoes are transmitting, or (3) all mosquitoes are transmitting. A more advanced approach has been applied by Paaijmans et al. [25] that considers the fact that even after long incubation periods not all mosquitoes of a batch are able to transmit the virus. Here, we decided to use the time until the first observed occurrence of transmission or infection of the SG for the data shown in Figures 1 and 2 for two reasons. First, this is the most conservative approach, as it utilizes the shortest possible EIP and hence is unlikely to underestimate risk. Second, in some cases batches consisted of only five or fewer mosquitoes [15]–[17], which is too few to derive statistically meaningful fractions. In order to facilitate the application of advanced statistical methods, this issue should be taken into account during the design of future experiments: in our opinion, batches of 20 to 30 mosquitoes, as used by Salazar et al. [19] and Paaijmans et al. [25], are desirable.

Another important issue to note is that past laboratory studies usually held temperatures constant over the whole experiment. This neglects the fact that in nature diurnal temperature is far from constant. Recent studies imply that diurnal fluctuations in temperature may play a more decisive role for pathogen amplification than previously thought [26], [27]. Including thermal fluctuations in future experiments and comparing the results with those from identical experiments with constant temperatures may prove rewarding.

Furthermore, not only the current main vector of dengue, *A. aegypti*, deserves attention: *A. albopictus* has undergone a vast global spread over the last decades [28] and is being considered as serving as a potential future main vector of dengue in Europe [29]. Until recently, knowledge about the EIP of dengue for *A. albopictus* was scarce and was mentioned only in a side note in the study by McLean et al. stating that “comparable results were obtained with…*A. albopictus* mosquitoes” [16]. In 2012, Richards et al. compared the vector competence of *A. albopictus* and *A. aegypti* for dengue at different temperatures [30]. Even though the duration of the EIP was not explicitly examined (a fixed incubation period of 14 days was used), this study can be regarded as a step in the right direction, since experiments focusing on *A. albopictus* are urgently needed.

In conclusion, further studies on the EIP of dengue based on experiments with modern methodology and adequately high resolution in time and temperature may facilitate risk assessment by improving mechanistic as well as correlative modeling approaches. Since the lack of knowledge on the temperature dependence of the EIP seems to be even bigger when it comes to other arthropod-borne viral diseases such as Chikungunya, the identified challenges and suggestions may turn out to be of relevance beyond the example of dengue.

## Supporting Information

Table S1 **Summary of the data obtained from the literature.** This table provides information about the different experimental studies, including study material used and methodological details. The duration until the first observed transmission or infection of SG at a given temperature is given for each study.
(XLS)

## Abbreviations

EIP: extrinsic incubation period
SG: salivary glands
